# The Impact of Induced Optical Blur on Monocular and Binocular Depth-Related Visuomotor Task Performance

**DOI:** 10.1167/iovs.66.15.8

**Published:** 2025-12-02

**Authors:** Tai Jarkum, Preetirupa Devi, Joshua A. Solomon, Christopher W. Tyler, Shrikant R. Bharadwaj

**Affiliations:** 1 Brien Holden Institute of Optometry and Vision Sciences, LV Prasad Eye Institute, Banjara Hills, Hyderabad, Telangana, India; 2 Professor Brien Holden Eye Research Centre, Hyderabad Eye Research Foundation, LV Prasad Eye Institute, Banjara Hills, Hyderabad, Telangana, India; 3 Centre for Applied Vision Research, City St. George's, University of London, Northampton Square, London, United Kingdom

**Keywords:** anisometropia, astigmatism, blur, defocus, stereopsis, visuomotor

## Abstract

**Purpose:**

The purpose of this study was to determine the impact of induced optical blur on a 3D task that probes complex visuomotor performance capabilities of humans.

**Methods:**

Fifteen visually normal, cyclopleged adults (mean ± 1 SD = 23 ± 2.6 years) guided a metal loop along a wire convoluted in depth without making contact, while being video recorded for analysis. The task was performed binocularly and monocularly, without blur, and with two magnitudes of induced spherical and astigmatic blur of equal strengths (2.25 diopters [D] and 6.25 D). Blur patterns were induced before both eyes (isometropia) or before only one eye (anisometropia). For isometropic astigmatism, blur was also induced with parallel and orthogonal axes in both eyes. The buzz-wire patterns, viewing condition, and induced blur were all randomized across participants.

**Results:**

Binocular error rate (number of loop-to-wire contacts per second) and error duration (percentage of time spent making errors) increased at high blur strength (*P* < 0.001), more so for astigmatism than spherical power (*p* < 0.001) and more so for isometropic than anisometropic viewing (*P* = 0.02). Low astigmatism with orthogonal axes bilaterally produced higher error rate and error duration than astigmatism with parallel axes bilaterally (*P* < 0.001). Only error duration increased with high blur for monocular viewing (*P* ≤ 0.004). Task speed remained invariant across test conditions. Multiple repetitions did not impact task performance.

**Conclusions:**

The deterioration of depth-related visuomotor task performance with optical blur depends on its magnitude, radial symmetry and the similarity between the two eyes. Performance drop is largely from spending more time making/correcting errors, whereas the overall speed remained undiminished.

Day-to-day activities like inserting a key into a keyhole or pouring water from a jug into a container are essential visuomotor tasks that require accurate estimates of 3D depth. The hand actions associated with these tasks may be guided by binocular retinal disparity plus monocular depth cues (e.g. motion parallax and texture), with the weight assigned to the former cue being larger than the latter ones.^1^^,^^2^ Two studies from Devi et al. support this notion using a visuomotor task that requires participants to move a loop around a wire convoluted in depth without contact.^3^^,^^4^ Error rates in this task increase with the loss of binocularity,^3^ and the associated binocular advantages (i.e. the extent to which binocular error rates are lower than monocular values) decline when binocularity is compromised due to blurred vision from distorted optics.^3^^,^^4^ Task speed also decreases with absent/degraded binocularity, albeit with a smaller effect size than that of error rates.^3^^,^^4^ Systematically investigating the impact of blurred vision on depth-related task performance is the primary goal of the present study.

Retinal image blur may impact depth-related visuomotor task performance for two reasons. First, optical blur limits visual resolution by degrading contrast and inducing phase shifts in the retinal image.^5^^–^^7^ Both factors impair the ability to resolve the critical details required to perform the task (e.g. estimating the diastereoptic gap between the loop and wire in the buzz-wire task; Fig. 1). Second, dissimilar blur in the two eyes impacts binocular processing by impairing correspondence matching in the monocular retinal images,^8^^,^^9^ reducing the overall quality of the disparity signal^8^ and suppressing the worse eye.^10^^–^^13^ Finally, the gains of vergence-related eye movements and ocular accommodation also decrease with blur, thus impairing the experience of clear and single binocular vision.^14^^,^^15^ All these factors may ultimately limit the stereoscopic depth and diastereopsis calculations required by visuomotor tasks (Fig. 1). In the context of the Devi et al. (2025) study described above,^4^ the exaggerated wavefront aberrations arising from distorted optics of the eye translate into significant, radially asymmetric retinal blur profiles.^18^^,^^19^ The blur profiles may also be dissimilar in the two eyes due to asymmetric disease severity.^18^^,^^19^ All of these factors could have influenced the buzz-wire task performance in that study.^4^ That there may be complex interactions between these blur dimensions to determine visuomotor task performance is also suggested by differences in the results obtained between eyes with distorted optics (keratoconus) and with regular refractive errors (uncorrected myopia) in their study.^4^ The myopic cohort, characterized primarily by isometropic, spherical blur profiles, continued to show a binocular advantage in error rates, whereas the keratoconic cohort, characterized by complex blur profiles as described above, lost the binocular advantage.^4^

**Figure 1.**
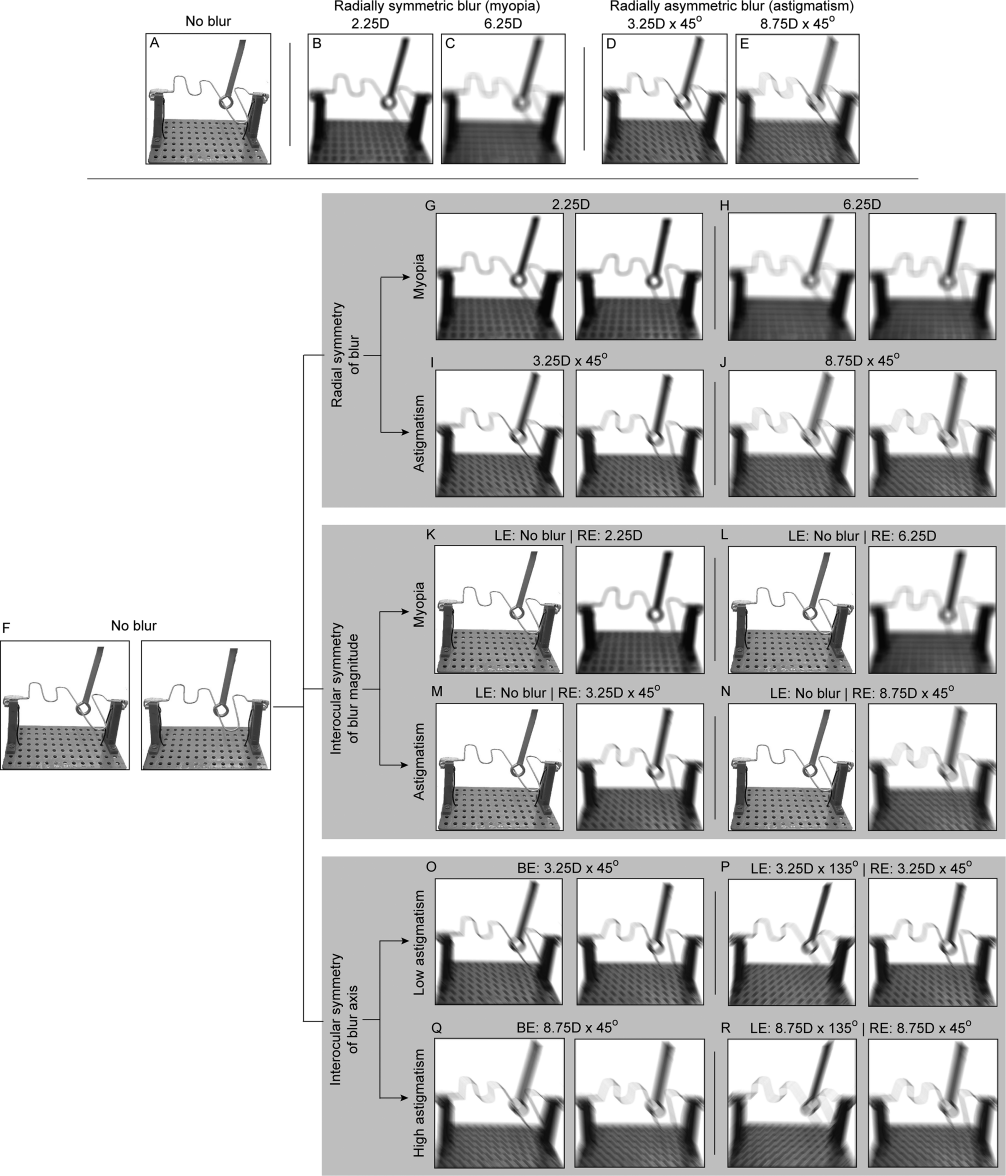
Point-of-view simulations of the buzz-wire apparatus with clear vision (**A**) and with different patterns of monocular optical blur (**B–E**). **F–R** Shows simulated cross-fusible stereo image pairs of the different binocular viewing conditions in this study. All optical simulations were generated for 555 nm light and 5 mm pupil diameter, using standard Fourier optics techniques.^16^ The simulations were created by convolving the point-of-view images of buzz-wire with the point spread function obtained by inducing a specific blur along with the population-averaged higher-order Zernike wavefront aberrations reported by Cheng et al. (2004).^17^

A systematic study is required to tease out the individual and combined contributions of these blur factors on the buzz-wire task performance. Few studies in the literature have investigated how vision loss from induced optical^20^^,^^21^ and non-optical (Bangerter filter)^22^ blur affects depth-related visuomotor tasks like bead threading, water pouring, peg placement, and sports-related interceptive actions. In general, these studies show worsening of task performance with increasing magnitudes of blur. Some tasks like bead threading appear to be more vulnerable to optical blur compared with others like the water pouring task.^21^ Although, in principle, these results demonstrate the negative impact of induced blur on visuomotor tasks, the relative impacts of different blur dimensions described above on such tasks remains unknown. This knowledge gap was addressed in the present study by systematically investigating the impact of two different magnitudes of spherical and astigmatic blur presented isometropically or anisometropically on the monocular and binocular buzz-wire task performance (see Fig. 1). The following hypotheses were tested here.

1. Monocular and binocular buzz-wire task performances will worsen with induced blur, relative to the no blur condition (see Figs. 1A versus 1B–E, Figs. 1F versus 1G–N). This will be for the aforesaid reasons of loss in visual resolution and binocularity.
2. Astigmatism will produce greater loss of task performance than comparable strengths of spherical blur (see Figs. 1B, 1C versus 1D, 1E, respectively). This will be because meridional blur in astigmatism may cause greater difficulty in diastereoptic judgments relative to the uniform image-quality loss with spherical blur (see Figs. 1B, 1C versus 1D, 1E, respectively). Astigmatism also tends to produce a larger subjective blurring effect than spherical blur.^23^^,^^24^
3. Anisometropia will produce greater loss of binocular task performance than comparable magnitudes of isometropia (see Figs. 1G, 1H versus 1K, 1L, respectively, and Figs. 1I, 1J versus 1M, 1N, respectively). This will be for the aforesaid reasons of binocularity loss with unequal magnitudes of blur in the two eyes.^8^^–^^13^
4. Astigmatism with orthogonal axes in the two eyes will result in greater loss of task performance than those with parallel axes in the two eyes (see Figs. 1O, 1Q versus 1P, 1R). This will be for the reason of binocular correspondence matching.
5. The binocular advantage of task performance will deteriorate in the presence of all forms of blur profiles owing to the underlying loss of binocularity, relative to the no blur condition.

## Methods

### Participants

The study adhered to the tenets of the Declaration of Helsinki, and it was approved by the Institutional Review Board of LV Prasad Eye Institute (LVPEI), Hyderabad, India. The experiment was initiated after all participants signed the written consent form. Fifteen participants (mean ± 1 SD age = 23 ± 2.6 years), based on convenient sampling, were recruited for the study whose uncorrected, monocular distance visual acuity was better than or equal to 20/25 in both eyes, spherical equivalent refractive error was ≤ ±0.50 D in both eyes, stereoacuity better than or equal to 40 arc sec and they were free of any ocular or binocular vision anomalies.

### The Apparatus, Task, and Outcome Measures

**Figure 2.**
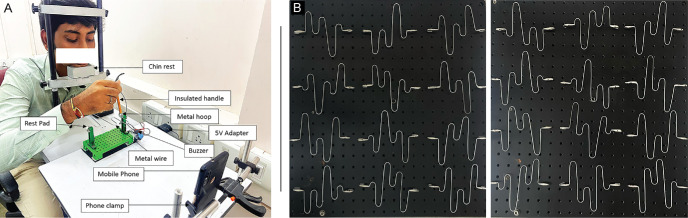
(**A**) The experimental set-up with the key elements highlighted. (**B**) The profiles of the 24 different buzz-wire patterns used in the experiment.

The buzz-wire task involves passing a metallic loop around the wire pattern convoluted in depth, without contact (Fig. 2A). Physical contact between the loop and the wire results in an auditory “buzz,” signaling an error in the task. A total of 24 unique buzz-wire patterns with 5 to 6 depth modulations of 6.5 cm, 4.0 cm, and 1.0 cm from the base position across the entire wire length (40.8 cm) were created to avoid practice effects (Fig. 2B). This ensured that a given pattern was used no more than twice across the entire experiment. The participant's head was stabilized using a chin and forehead rest at the beginning of the experiment, ensuring that the distance between the participants and the buzz-wire setup was approximately 33 cm. Stabilizing the head also ensured that the pattern of astigmatic blur experienced did not vary during the task. The task was performed 45 to 60 minutes after instillation of 1% cyclopentolate HCl eye drops to ensure that the induced blur profiles did not vary with the participant's accommodative behavior.^25^^,^^26^ The effect of cycloplegia was confirmed by near acuity worsening to >N8 on the standard near vision chart at 40 cm viewing distance. Additional eye drops were used, if necessary, to ensure that this criterion was met throughout the experiment. A near-correction of +3 D was placed before the participant's eyes to account for the 33 cm viewing distance at which the buzz-wire task was performed.

Task instructions and the process of data cleaning and analysis is described in Devi et al. (2024 and 2025).^3^^,^^4^ Task performance was quantified using three outcome variables. Error rate was calculated as the number of error buzzes over the total task duration (in errors/second). Error duration was calculated as the total time spent in error divided by the total task duration (in percentage). Speed was calculated as the length of the wire divided by the error-free time (in cm/second).

### The Induced Blur Conditions

The blur profiles included the two magnitudes of radially symmetric (spherical blur; see Figs. 1B, 1C) or asymmetric (astigmatic blur; see Figs. 1D, 1E) blur and with the blur being equal in the two eyes (isometropia; see Figs. 1G–J) or unequal in the two eyes (anisometropia; see Figs. 1K–N). Two variants of isometropic astigmatic blur were also tested — a profile with similar magnitude and axes of astigmatism in the two eyes (45 degrees) and a profile with similar magnitude but orthogonal axes of astigmatism in the two eyes (45 degrees in the right eye and 135 degrees in the left eye; see Figs. 1O–S). The impact of blur magnitude and radial symmetry on the buzz-wire task performance was investigated under binocular and monocular conditions. The impact of interocular symmetry was investigated only under binocular conditions. Monocular testing was always performed on the right eye with the left eye was occluded. For binocular viewing, the blur profiles were introduced either before both eyes (isometropic viewing) or only before the right eye (anisometropic viewing) while the left eye viewing remained unhindered. All blur profiles were induced using full-aperture trial lenses mounted on a trial frame at a 14 mm vertex distance. Each participant repeated the buzz-wire task thrice with each blur profile, resulting in a total of 48 repetitions per participant ([monocular baseline + 2 monocular spherical blur + 2 monocular astigmatic blur + binocular baseline + 2 isometropic spherical blur + 2 isometropic astigmatic blur with parallel axes in the 2 eyes + 2 isometropic astigmatic blur with orthogonal axes in the 2 eyes + 2 anisometropic spherical blur + 2 anisometropic astigmatic blur] × 3 repetitions of each condition = 48 trials). The first trial was always the binocular baseline condition, whereas the order of the remaining trials was randomized within and across participants to minimize any practice effect (see Supplement II for the control experiment investigating the impact of practice on the buzz-wire task performance). Data were collected across 2 days on each participant, averaging 2.5 hours per participant per day. Short breaks were provided between trials or whenever required to reduce fatigue.

The two levels of optical blur were purposely chosen in this study to induce significant loss of visual resolution and binocularity (see Supplement I for an investigation of the relationship between different blur magnitudes and buzz-wire task performance). Like Piano and O'Connor (2013),^13^ the lower magnitude of spherical blur (2.25 D) used here resulted in an 8-line loss of visual acuity from baseline (mean ± 1 SD visual acuity across 8 participants = 0.82 ± 0.16 logMAR), whereas the higher magnitude (6.25 D) resulted in a 14-line loss of visual acuity from baseline (1.42 ± 0.18 logMAR units). All visual acuities were measured under photopic conditions using a computerized logMAR optotype presentation system (COMPlog Vision Measurement, London, UK).^27^ Herein, five Sloan optotypes were randomly displayed on an LCD screen and their angular subtense decreased using a staircase algorithm until three of five optotypes were incorrectly identified. LogMAR acuity was recorded as the number of optotypes correctly identified at termination, with 0.02 logMAR units allotted per optotypes. Comparable strengths of astigmatic blur were induced using cylindrical lenses at 45-degree axes. Note that the total blur strength of a spherocylindrical lens is:

$$B=\sqrt{{\left(S\;+\frac{C}{2}\right)}^{2}+{\left(-\frac{C}{2}\cos2\beta \right)}^{2}+{\left(-\frac{C}{2}\sin2\beta \right)}^{2}},$$

where *S* is the power of the spherical component, *C* is the power of the (positive) cylindrical component, and β is the cylindrical axis.^28^ The cylindrical power of our low-powered and high-powered lenses at 45 degree axis were 3.25 D and 8.75 D, respectively. These cylindrical powers were selected as the closest available strengths matching the 2 spherical powers of 2.25 D and 6.25 D according to the equivalence formula described above by Thibos et al. (1997).^28^ The specific values of matched blur strengths were 2.30 D and 6.19 D at axis 45 degrees. Use of these lenses resulted in 7-line and 12-line acuity losses, relative to baseline, in the same 8 participants (logMAR values were of 0.76 ± 0.15 and 1.25 ± 0.15, respectively).

In addition to the buzz-wire task, stereo perception thresholds were also measured under cycloplegia (but corrected for the test viewing distance), at a 50-cm viewing distance, using the technique described by Devi et al. (2025).^4^ Random-dot stimuli were presented on an LCD monitor and controlled using the Psychtoolbox-3 interface of MATLAB (R2024a; The MathWorks, Natick, MA, USA). These dichoptic stimuli were fused using a handheld stereo viewer with built-in periscopic mirrors to adjust for the participant's horizontal phoria and interpupillary distance (Screen-Vu Stereoscope, Portland, OR, USA). The cyclopean image was a vertically oriented rectangular bar tilted either to the left or to the right in uncrossed horizontal retinal disparity. Participants indicated the direction of the bar tilt while the retinal disparity varied in a 2-down and 1-up adaptive staircase with each presentation for 11 reversals. Whereas all participants had clinical stereo thresholds better than 40 arc sec (measured using Wirt circles), the average (±1 SEM) psychophysical stereo threshold (measured with random-dot stimuli) for the baseline condition was 102 ± 19 arc sec. This difference may be attributed to the nature of the stereo stimuli as well as cycloplegia in the laboratory.^29^ Stereo thresholds worsened to ≥500 arc sec across all induced blur conditions. Because the stereo thresholds were found to have limited correlation with the buzz-wire task performance in the Devi et al. (2025)^4^ study, no further analyses of these thresholds are performed here. Instead, these data simply serve as evidence for deteriorated sensory binocularity across all the induced blur conditions in the present study.

### Statistical Analyses

Matlab and SPSS (version 27; IBM, SPSS Inc., Armonk, NY, USA) were used for data analyses. The Shapiro-Wilk test revealed no significant departure from normality in the three outcome variables and hence the data trends were described using parametric statistics. Several statistical analyses were performed to gain a comprehensive understanding of the impact of different combinations of blur and viewing conditions on the outcome variables of the buzz-wire task. These details are shown in Table 1, categorized by the underlying study hypotheses. Hypothesis testing also involved an analysis of the binocular advantage in task performance for all three outcome measures (see Table 1). The binocular advantages in the error rate and error duration were calculated as ratios of monocular performance to binocular performance. The binocular advantage in speed was calculated as the ratio of binocular speed to monocular speed. These calculations ensured that a ratio greater than unity indicated superior performance under binocular than monocular viewing. For the isometropic blur condition, the monocular performance with the corresponding value of blur was used to compute the binocular advantage. For the anisometropic blur condition, the monocular performance without any induced blur was used to compute the binocular advantage. This was done under the assumption that the eye with the clear vision is used for viewing, whereas the fellow eye with blurred vision may be suppressed in anisometropia.^30^^,^^31^ For the parallel versus orthogonal axes of astigmatism, the monocular performance with corresponding value of blur at 45 degrees and 135 degrees axis was used to compute the binocular advantage.

**Table 1.** Description of the Different Statistical Analyses Performed to Test the Study Hypotheses

	Statistics	Independent Factors	Dependent Variables	Text Reference
***Hypotheses 1 and 5: Impact of induced blur on task performance versus baseline***				
Mono viewing	1-factor RM-MANOVA	Baseline and all monocular induced blur conditions	Error rate, error duration, and speed	Table 2, Section 2
Bino viewing		Baseline and all binocular induced blur conditions		Table 2, Section 3
Bino advantage				Table 2, Section 4
***Hypotheses 2 and 3: Impact of radial and interocular symmetry of blur on task performance***				
Mono viewing	2-factor RM-MANOVA	Blur magnitude and radial symmetry of blur	Error rate, error duration, and speed	Table 3, Section 1
Bino viewing	3-factor RM-MANOVA	Blur magnitude, radial symmetry, and interocular symmetry of blur		Table 3, Section 2
Bino advantage	3-factor RM-MANOVA			Table 3, Section 3
***Hypothesis 4: Impact of parallel versus orthogonal astigmatic axis on task performance***				
Bino viewing	2-factor RM-MANOVA	Blur magnitude and astigmatic axis orientation	Error rate, error duration, and speed	Table 4, Section 1
Bino advantage	2-factor RM-MANOVA			Table 4, Section 2

RM-MANOVA, Repeated Measures Multiple Analysis of Variance.

The column “Text reference” indicates the location in the tables where the results of a particular statistical analysis appear in the text.

## Results

**Figure 3.**
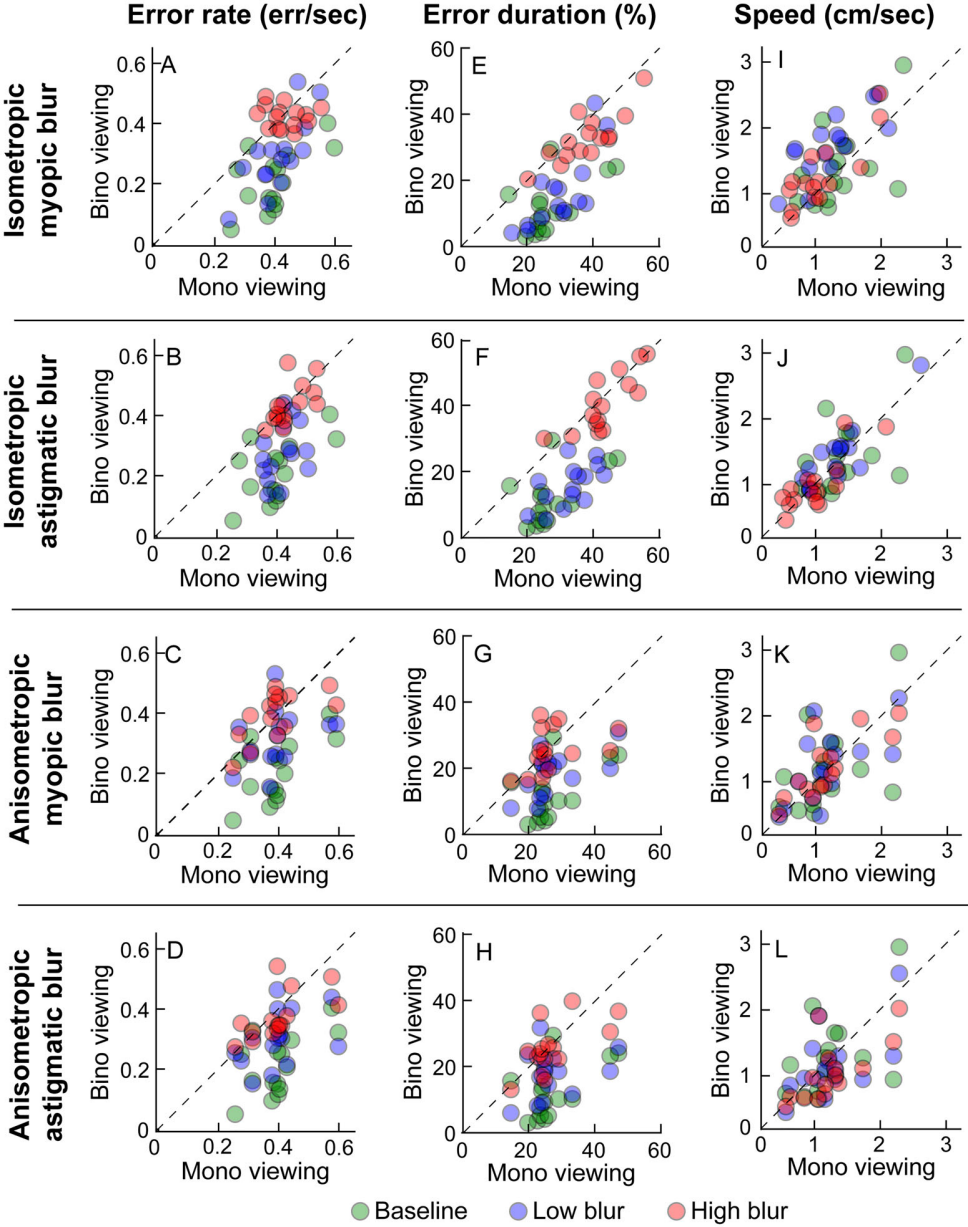
Scatter diagrams of binocular and monocular error rate (**A–D**), error duration (**E–H**), and speed (**I–L**) under baseline no-blur condition (*green circles*), low blur (*blue circles*), and high blur (*red circles*) viewing conditions. The *top two rows* show data for isometropic blur and the *bottom two rows* show equivalent data for anisometropic blur. The same baseline data are plotted in each panel for ease of comparison. The *dashed diagonal line* in each panel represents equal binocular and monocular performance.

**Figure 4.**
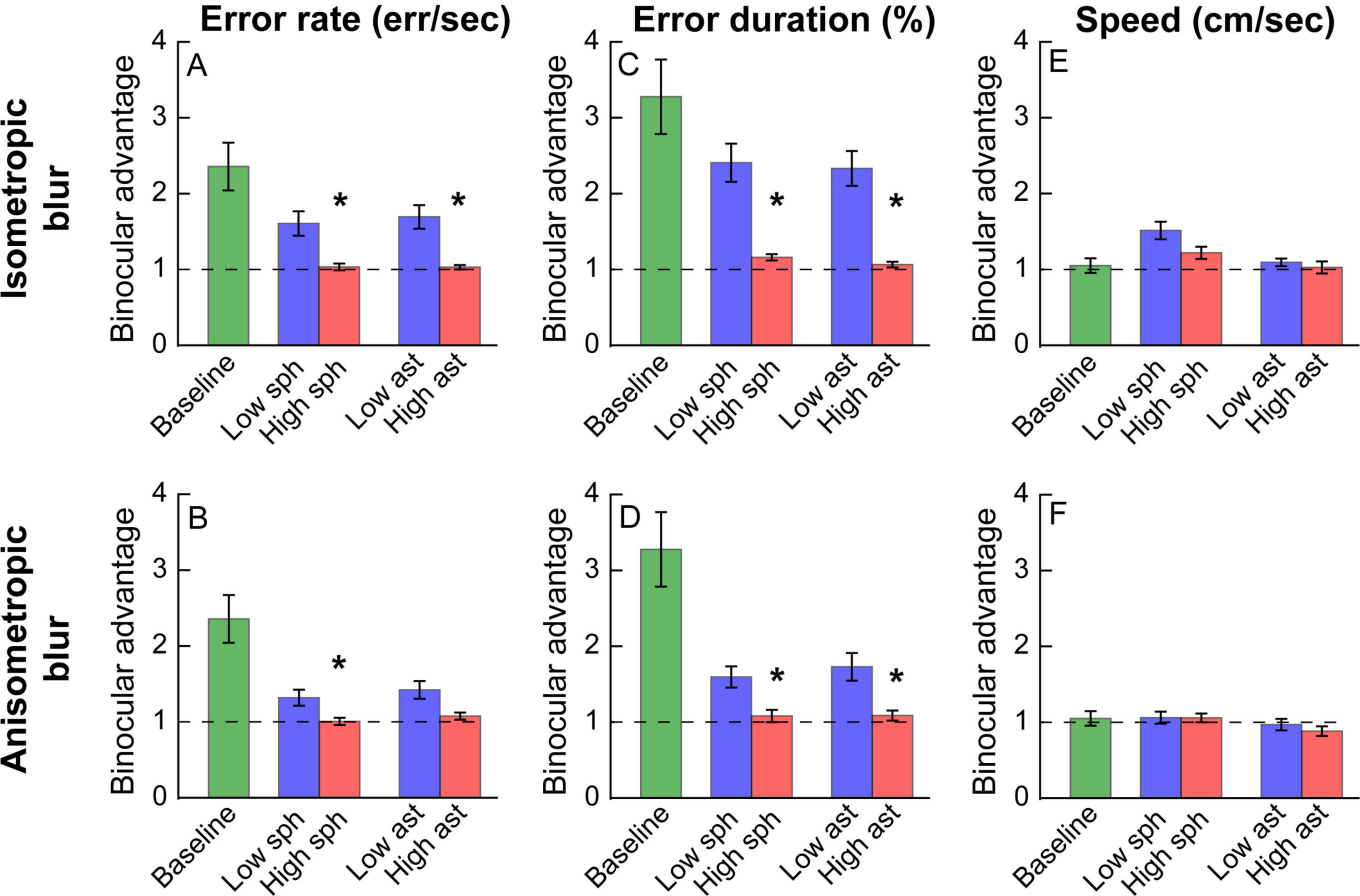
Mean ± 1 SEM binocular advantage in error rate (**A**, **B**), error duration (**C**, **D**), and speed (**E**, **F**), under baseline no-blur condition (*green bars*), low blur (*blue bars*), and high blur (*red bars*) viewing conditions. The *dashed horizontal line* in each panel indicates the level of no binocular advantage. The baseline data are the same between isometropic and anisometropic blur conditions. The *asterisk* denotes the blur conditions that were significantly different (*P* < 0.05) from baseline.

Figures 3, 4 show the binocular and monocular outcome variables and the respective binocular advantages for the different conditions tested in this study. The data points for the baseline (no blur) and low blur conditions were below the line of equality for error rates and error durations, indicating superior performance under binocular viewing (see Fig. 3). The distribution of data points in the baseline and low blur condition overlapped, indicating no evidence for difference in task performance between these two conditions (see Fig. 3). On the other hand, the data distribution for the high induced blur condition shifted upward to the right in isometropia and simply upward in anisometropia, indicating increased error rates and error durations, relative to the other conditions (see Fig. 3, left and middle columns). Accordingly, the baseline viewing showed a robust binocular advantage for the two outcome variables in Figures 4A to 4D. This advantage was present but lower than the baseline condition for the low blur conditions, irrespective of radial or interocular symmetry (see Figs. 4A–D). There was no evidence for a binocular advantage in the high blur conditions (see Figs. 4A–D). Speed as an outcome parameter did not indicate any specific trend, regardless of the blur conditions.

### Impact of Induced Blur on Buzz-Wire Task Performance, Relative to Baseline Viewing

The one-factor RM-MANOVA showed a significant main effect of induced blur on the monocular and binocular task performance (*P* < 0.001) and on the binocular advantage of task performance (*P* < 0.001). For monocular viewing, Bonferroni-corrected pairwise comparison revealed significant worsening of task performance from the baseline condition only for error duration with high spherical and astigmatic blur (Table 2, Section 2). For binocular viewing, the error rates and error durations were significantly higher than the baseline condition for high spherical and astigmatic blur under isometropic and anisometropic viewing conditions (see Table 2, Section 3). Similarly, the binocular advantage for error rate and error duration was also significantly lower than the baseline condition for only the high spherical and astigmatic blur under isometropic and anisometropic viewing conditions (see Table 2, Section 4).

**Table 2.** Baseline Parameters of Error Rate, Error Duration, and Speed Under Binocular and Monocular Viewing Condition (Section 1)

	Error Rate (Err/Sec)		Error Duration (%)		Speed (cm/Sec)	
	Mean ± SEM	*P* Value	Mean ± SEM	*P* Value	Mean ± SEM	*P* Value
Section 1: Baseline Parameters						
Monocular	0.39 ± 0.02	**<0.001**	26.97 ± 2.22	**<0.001**	1.37 ± 0.11	0.47
Binocular	0.20 ± 0.02		11.81 ± 2.10		1.38 ± 0.14	
	**Error Rate (Err/Sec)**		**Error Duration (%)**		**Speed (cm/Sec)**	
	**Mean diff ± SEM**	***P* Value**	**Mean diff ± SEM**	***P* Value**	**Mean diff ± SEM**	***P* Value**

Section 2: Monocular viewing						
Low sph	−0.01 ± 0.02	>0.99	−2.89 ± 1.99	>0.99	0.14 ± 0.10	>0.99
High sph	−0.04 ± 0.27	>0.99	−10.82 ± 2.36	**0.004**	0.28 ± 0.10	0.18
Low astig	−0.02 ± 0.02	>0.99	−5.63 ± 1.82	0.79	0.12 ± 0.08	>0.99
High astig	−0.04 ± 0.02	0.92	−16.03 ± 1.62	**<0.001**	0.42 ± 0.12	**0.04**
Section 3: Binocular viewing						
Low sph iso	−0.08 ± 0.03	>0.99	−4.20 ± 2.80	>0.99	−0.34 ± 0.17	>0.99
High sph iso	−0.21 ± 0.02	**<0.001**	−20.91 ± 1.90	**<0.001**	0.10 ± 0.16	>0.99
Low astig iso	−0.06 ± 0.03	>0.99	−4.11 ± 1.87	>0.99	0.04 ± 0.10	>0.99
High astig iso	−0.22 ± 0.03	**<0.001**	−29.06 ± 2.08	**<0.001**	0.46 ± 0.16	0.43
Low sph aniso	−0.11 ± 0.03	**0.03**	−6.52 ± 2.15	0.32	−0.03 ± 0.13	>0.99
High sph aniso	−0.18 ± 0.03	**<0.001**	−13.91 ± 2.30	**<0.001**	−0.03 ± 0.15	>0.99
Low astig aniso	−0.09 ± 0.03	0.46	−5.97 ± 2.62	>0.99	0.10 ± 0.12	>0.99
High astig aniso	−0.16 ± 0.03	**<0.001**	−13.72 ± 2.67	**0.01**	0.21 ± 0.14	>0.99
Section 4: Binocular advantage						
Low sph iso	0.75 ± 0.30	0.91	0.87 ± 0.48	>0.99	−0.46 ± 0.16	0.44
High sph iso	1.32 ± 0.31	**0.02**	2.12 ± 0.49	**0.02**	−0.17 ± 0.12	>0.99
Low astig iso	0.66 ± 0.31	1.00	0.95 ± 0.48	>0.99	−0.04 ± 0.10	>0.99
High astig iso	1.32 ± 0.32	**0.03**	2.21 ± 0.49	**0.02**	0.02 ± 0.14	>0.99
Low sph aniso	1.03 ± 0.29	0.09	1.68 ± 0.48	0.12	−0.01 ± 0.10	>0.99
High sph aniso	1.35 ± 0.32	**0.03**	2.19 ± 0.50	**0.02**	−0.01 ± 0.11	>0.99
Low astig aniso	0.93 ± 0.35	0.61	1.55 ± 0.56	0.53	0.08 ± 0.10	>0.99
High astig aniso	1.28 ± 0.32	0.05	2.19 ± 0.53	**0.03**	0.17 ± 0.11	>0.99

iso, isometropic; aniso, anisometropic; astig, astigmatism; sph, spherical.

Negative values of the mean difference indicate increased error rate, error duration, and speed with induced blur, relative to baseline viewing. Sections 1 and 2 of this table show the results for monocular and binocular viewing, respectively. Section 3 shows the results for binocular advantage. Comparisons that reached significance at *P* ≤ 0.05 are indicated in bold.

Results of the post hoc Bonferroni test conducted as part of the one-factor RM–MANOVA analysis to compare the error rate, error duration, and speed under baseline and the different induced blur conditions (Sections 2–4).

### Impact of Radial and Bilateral Symmetry of Blur on Buzz-Wire Performance

The two-factor RM-MANOVA for monocular viewing revealed significant main effects of blur magnitude and radial symmetry across all three outcome variables (Table 3, Section 1a). Univariate analyses revealed significant effects of blur magnitude and radial symmetry only for the error duration (see Table 3, Section 1b) and a significant effect of blur magnitude on the speed (see Table 3, Section 1b). The three-factor RM-MANOVA revealed significant main effect of all the three factors (blur magnitude, radial symmetry of blur, and interocular symmetry of blur) on binocular task performance (see Table 3, Section 2a). Significant interactions were also noted between the factors, indicating that the impact of these factors on the binocular buzz-wire task performance is not independent of each other (see Table 3, Section 2a). The univariate analyses indicated significant main effects and interactions for the error duration variable (see Table 3, Section 2b). Only sporadic factors were significant for error rates and speed, as highlighted in Table 3, Section 2b.

**Table 3.** Results of Two-Factor and Three-Factor RM-MANOVAs Performed to Determine the Effect of Different Patterns of Induced Blur on the Monocular and Binocular Task Performance, Respectively

Section 1: Two–Factor RM–MANOVA for Monocular Performance									
1a. Multivariate Tests									
			F		*P* Value			Partial ƞ^2^	
Blur magnitude			13.56		**<0.001**			0.77	
Radial symmetry			4.81		**0.02**			0.54	
Blur magnitude × radial symmetry			1.34		0.30			0.25	
**1b. Univariate Tests**									

	**Error Rate (Err/sec)**			**Error Duration (%)**			**Speed (cm/sec)**		
	**Mean ± SEM**	***P* Value**	**Partial ƞ^2^**	**Mean ± SEM**	***P* Value**	**Partial ƞ^2^**	**Mean ± SEM**	***P* Value**	**Partial ƞ^2^**

Blur magnitude									
Low	0.41 ± 0.01	0.1	0.17	31.23 ± 1.78	**<0.001**	0.74	1.24 ± 0.11	**0.001**	0.53
High	0.43 ± 0.01			40.39 ± 2.02			1.02 ± 0.10		
Radial symmetry									
Spherical	0.42 ± 0.01	0.54	0.02	33.82 ± 1.96	**0.006**	0.42	1.16 ± 0.11	0.37	0.05
Astigmatic	0.42 ± 0.01			37.80 ± 1.77			1.10 ± 0.10		
Blur magnitude × radial symmetry	–	0.93	0.0001	–	0.27	0.08	–	0.09	0.19
**Section 2: Three–Factor RM–MANOVA for Binocular Performance**									
**2a. Multivariate Tests**									

			**F**		***P* Value**			**Partial ƞ^2^**	

Blur magnitude			75.81		**<0.001**			0.95	
Radial symmetry			13.46		**<0.001**			0.77	
Interocular symmetry			4.78		**0.02**			0.54	
Blur magnitude × radial symmetry			4.13		**0.03**			0.5	
Radial symmetry × interocular symmetry			5.78		**0.01**			0.6	
Blur magnitude × interocular symmetry			47.3		**<0.001**			0.92	
All interactions			1.2		0.35			0.23	
**2b. Univariate Tests**									

	**Error Rate (Err/sec)**			**Error Duration (%)**			**Speed (cm/sec)**		
	**Mean ± SEM**	***P* Value**	**Partial ƞ^2^**	**Mean ± SEM**	***P* Value**	**Partial ƞ^2^**	**Mean ± SEM**	***P* Value**	**Partial ƞ^2^**

Blur magnitude									
Low	0.30 ± 0.02	**<0.001**	0.81	17.02 ± 1.64	**<0.001**	0.94	1.45 ± 0.11	**<0.001**	0.62
High	0.41 ± 0.01			31.22 ± 1.63			1.21 ± 0.10		
Radial symmetry									
Spherical	0.36 ± 0.02	0.15	1.42	23.2 ± 1.68	**0.03**	0.29	1.46 ± 0.11	**0.001**	0.56
Astigmatic	0.34 ± 0.02			25.03 ± 1.56			1.19 ± 0.11		
Interocular symmetry									
Isometropia	0.36 ± 0.02	0.47	0.04	26.39 ± 1.91	**0.004**	0.45	1.33 ± 0.11	0.96	0.0001
Anisometropia	0.35 ± 0.02			21.85 ± 1.48			1.39 ± 0.11		
Blur magnitude × radial symmetry	–	0.70	0.01	–	**0.03**	0.28	–	0.64	0.01
Blur magnitude × interocular symmetry	–	**0.01**	0.37	–	**<0.001**	0.87	–	**<0.001**	0.62
Radial symmetry × interocular symmetry	–	0.17	0.13	–	**0.04**	0.27	–	**0.004**	0.47
All interactions	–	0.43	0.04	–	0.06	0.22	–	0.36	0.05
**Section 3: Three–Factor RM–MANOVA for Binocular Advantage**									
**3a. Multivariate Tests**									

			**F**		***P* Value**			**Partial ƞ^2^**	

Blur magnitude			12.72		**<0.001**			0.76	
Radial symmetry			4.73		**0.02**			0.54	
Interocular symmetry			6.75		**0.006**			0.63	
Blur magnitude × radial symmetry			0.35		0.79			0.08	
Radial symmetry × interocular symmetry			1.14		0.37			0.22	
Blur magnitude × interocular symmetry			5.24		**0.02**			0.56	
All interactions			0.88		0.48			0.18	
**3b. Univariate Tests**									

	**Error Rate (Err/sec)**			**Error Duration (%)**			**Speed (cm/sec)**		
	**Mean ± SEM**	***P* Value**	**Partial ƞ^2^**	**Mean ± SEM**	***P* Value**	**Partial ƞ^2^**	**Mean ± SEM**	***P* Value**	**Partial ƞ^2^**

Blur magnitude									
Low	1.51 ± 0.08	**<0.001**	0.73	2.02 ± 0.14	**<0.001**	0.74	1.16 ± 0.04	**0.03**	0.28
High	1.04 ± 0.02			1.10 ± 0.04			1.05 ± 0.04		
Radial symmetry									
Spherical	1.24 ± 0.05	0.29	0.08	1.56 ± 0.08	0.92	0.001	1.21 ± 0.04	**0.002**	0.51
Astigmatic	1.30 ± 0.06			1.55 ± 0.09			0.99 ± 0.05		
Interocular symmetry									
Isometropia	1.34 ± 0.06	0.15	0.14	1.74 ± 0.10	**0.007**	0.41	1.21 ± 0.04	**0.02**	0.33
Anisometropia	1.21 ± 0.07			1.37 ± 0.09			0.99 ± 0.06		
Blur magnitude × radial symmetry	–	0.60	0.02	–	0.69	0.01	–	0.32	0.07
Blur magnitude × interocular symmetry	–	0.05	0.25	–	**0.001**	0.53	–	0.16	0.14
Radial symmetry × interocular symmetry	–	0.68	0.01	–	0.33	0.07	–	0.08	0.20
All interactions	–	0.84	0.003	–	0.77	0.006	–	0.13	0.16

Sections 1 and 2 of this table show the results for monocular and binocular viewing, respectively. Section 3 of this table show the results for binocular advantage. Comparisons that reached statistical significance at *P* ≤ 0.05 are indicated in bold.

The three-factor RM-MANOVA also revealed significant main effects of all three factors on binocular advantage along with a significant interaction between blur magnitude and interocular symmetry (see Table 3, Section 3a). Univariate analyses showed a significant loss of binocular advantage in error rate only with blur magnitude (see Table 3, Section 3b). The binocular advantage in error duration significantly deteriorated for both blur magnitude and interocular symmetry of blur, with significant interaction between the two factors (see Table 3, Section 3b). The binocular advantage in speed also showed a significant loss with all these main factors (see Table 3, Section 3b).

**Figure 5.**
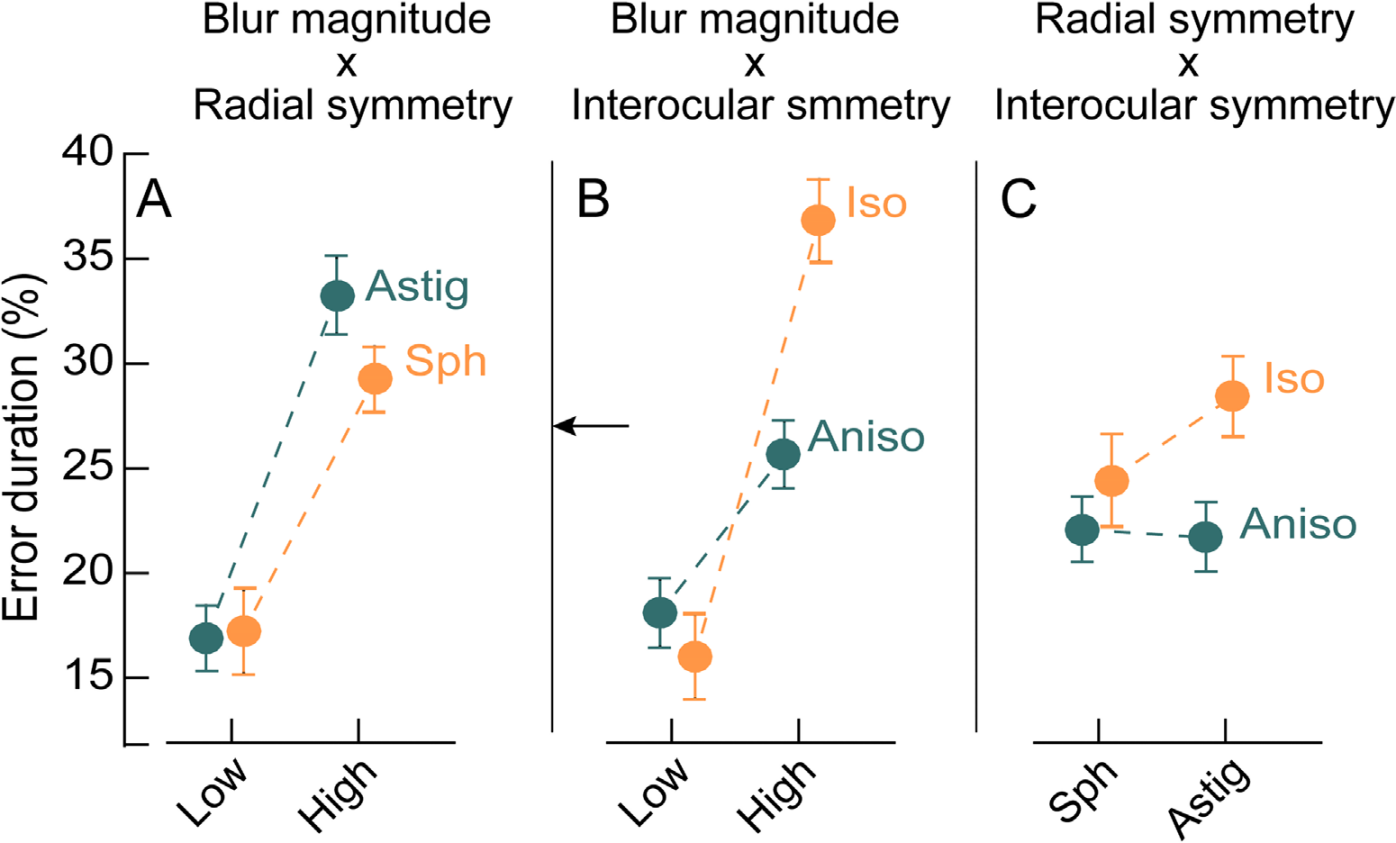
Impact of interactions among blur magnitude, radial symmetry, and interocular symmetry of blur on the mean ±1 SEM error duration in the binocular buzz-wire task. (**A**) Shows the interaction of blur magnitude and radial symmetry of blur for the combined isometropic and anisometropic blur conditions. (**B**) Shows the interaction of blur magnitude and interocular symmetry of blur for the combined spherical and astigmatic blur conditions. The horizontal arrow indicates the mean baseline (no blur) error duration for monocular viewing. (**C**) Shows the interaction of radial and interocular symmetry of blur for the combined low and high magnitudes of blur. The data points in each panel are connected only to highlight the interaction between the factors.

To better understand the nature of interactions among the different dimensions of blur, the error duration variable is plotted in Figure 5 for the different interaction elements shown in Table 3, Section 2b. Figure 5A plots the interaction between blur magnitude and its radial symmetry across the combined isometropic and anisometropic viewing conditions. The error durations were not statistically different for low spherical and astigmatic blur (t = 1.99, *P* = 0.06) but they were significantly higher for astigmatic than spherical blur at the high blur magnitude (t = 9.77, *P* < 0.001; see Fig. 5A). Figure 5B plots the interaction between blur magnitude and its interocular symmetry across the combined spherical and astigmatic viewing conditions. The error durations were not statistically different for low magnitudes of isometropic and anisometropic blur (t = −1.47, *P* = 0.15) but they were significantly higher for isometropic viewing than anisometropic viewing for the high magnitude of blur (t = 6.94, *P* < 0.001; see Fig. 5B). The mean (±1 SEM) error duration in the high anisometropic condition (25.63 ± 1.58%) also matched the mean error duration observed under the monocular baseline no blur viewing condition (26.97 ± 2.22%; see horizontal arrow location in Fig. 5B). The results indicate worse task performance with the high magnitude of isometropic than with a comparable level of anisometropic blur. Figure 5C plots the interaction between the radial and interocular symmetry of blur across the combined low and high blur magnitudes. Isometropic blur resulted in overall higher error durations than anisometropic blur, but this difference was greater for astigmatic than for spherical blur (t = 9.77, *P* <0.001; see Fig. 5C).

### Impact of the Bilateral Symmetry of Astigmatic Axis on Buzz-Wire Performance

**Figure 6.**
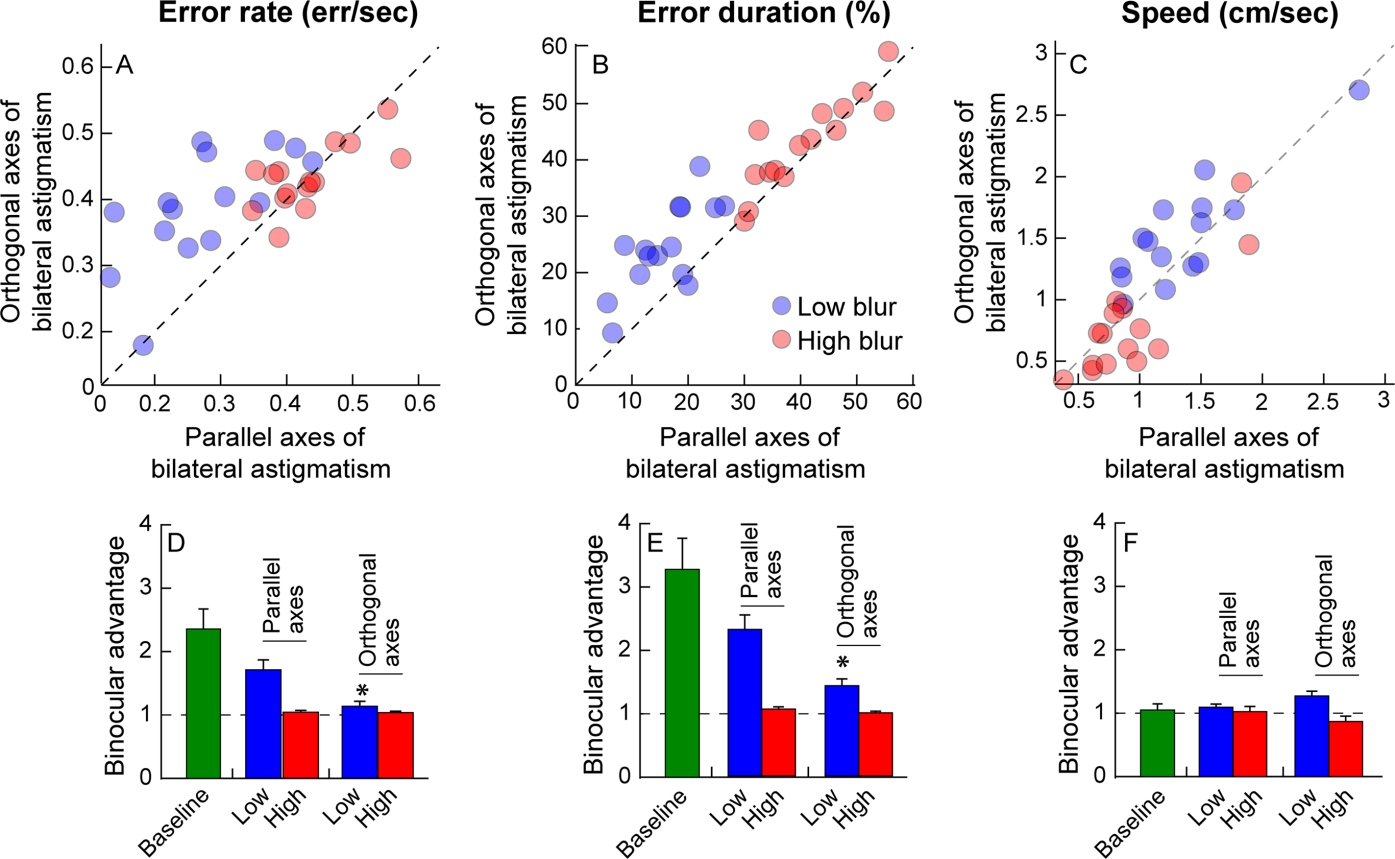
Outcome variables (as scatter diagrams in **A–C**) and binocular advantages (as bar graphs in **D–F**) with parallel and orthogonal axes of astigmatism in the two eyes under low and high blur viewing conditions. The *asterisk* symbols in panels **D** and **E** indicate significant differences between parallel and orthogonal axes.

Error rates (Fig. 6A) and error durations (Fig. 6B) were worse for the low blur condition with orthogonal axes orientation, relative to the parallel axes orientation (Fig. 6). This effect was absent for the high blur condition, with both sets of data falling along the line of equality (see Figs. 6A, 6B). Speed decreased with blur magnitude for both parallel and orthogonal axes orientations (see Fig. 6C). The two-factor RM-MANOVA revealed a significant main effect of blur magnitude and interocular astigmatic axis orientation and a significant interaction between the factors on the combined outcome variables (Table 4, Section 1a). Univariate tests revealed blur magnitude to have a significant effect on all three outcome variables, whereas the axis orientation had an effect only on the error rate and error duration (see Table 4, Section 1b). The binocular advantage for error rate (see Fig. 6D) and error duration (see Fig. 6E) decreased with astigmatic blur (see also Table 4, Section 2). It was completely lost when the magnitude of astigmatism was high, irrespective of its axis orientation (see Figs. 6D, 6E, Table 4, Section 2). Speed did not show strong trend in the binocular advantage (see Fig. 6F; Table 4, Section 2).

**Table 4.** Results of Two-Factor RM-MANOVA Performed to Determine the Impact of Astigmatic Axis Orientation on Binocular Buzz-Wire Task Performance

Section 1: Two–Factor RM–MANOVA for Binocular Performance									
1a. Multivariate Tests									
			F		*P* Value			Partial ƞ^2^	
Blur magnitude			14.13		**<0.001**			0.95	
Bilateral axes			87.85		**<0.001**			0.77	
Blur magnitude × bilateral axes			11.55		**0.001**			0.74	
**1b. Univariate Tests**									

	**Error Rate**			**Error Duration**			**Speed**		
	**Mean ± SEM**	***P* Value**	**Partial ƞ^2^**	**Mean ± SEM**	***P* Value**	**Partial ƞ^2^**	**Mean ± SEM**	***P* Value**	**Partial ƞ^2^**

Blur magnitude									
Low	0.33 ± 0.02	**<0.001**	0.61	20.15 ± 1.68	**<0.001**	0.95	1.44 ± 0.11	**<0.001**	0.75
High	0.43 ± 0.01			41.92 ± 2.10			0.86 ± 0.10		
Axis orientation									
Parallel	0.35 ± 0.02	**<0.001**	0.69	28.40 ± 1.83	**<0.001**	0.68	1.14 ± 0.10	0.62	0.01
Orthogonal	0.41 ± 0.01			33.67 ± 1.87			1.16 ± 0.10		
Blur magnitude × axis orientation	–	**<0.001**	0.61	–	**0.001**	0.54	–	**0.005**	0.43
**Section 2: Two–Factor RM–MANOVA for Binocular Advantage**									
**2a. Multivariate Tests**									

			**F**		***P* Value**			**Partial ƞ^2^**	
Blur magnitude			11.43		**0.001**			0.74	
Bilateral axes			7.45		**0.004**			0.65	
Blur magnitude × bilateral axes			10.94		**0.001**			0.73	
**2b. Univariate Tests**									

	**Error Rate**			**Error Duration?**			**Speed**		
	**Mean ± SEM**	***P* Value**	**Partial ƞ^2^**	**Mean ± SEM**	***P* Value**	**Partial ƞ^2^**	**Mean ± SEM**	***P* Value**	**Partial ƞ^2^**

Blur magnitude									
Low	1.41 ± 0.10	**0.001**	0.55	1.88 ± 0.15	**<0.001**	0.68	1.18 ± 0.95	**0.02**	0.31
High	1.03 ± 0.02			1.03 ± 0.03			0.947 ± 0.08		
Axis orientation									
Parallel	1.36 ± 0.09	**0.002**	0.52	1.70 ± 0.12	**<0.001**	0.60	1.06 ± 0.05	0.82	0.004
Orthogonal	1.07 ± 0.04			1.22 ± 0.06			1.07 ± 0.05		
Blur magnitude × axis orientation	–	**0.001**	0.53	–	**0.001**	0.56	–	**0.003**	0.49

## Discussion

### Summary of Results

Depth-related visuomotor task performance deteriorates in the presence of induced optical blur under binocular and monocular viewing conditions. The specific study results may be summarized as follows:

1) Error rates and error duration increased with induced optical blur under monocular and binocular viewing conditions, vis-à-vis, no blur viewing. Although this deterioration progressively increased with the magnitude of optical blur (see Supplement I), it reached statistical significance only with the high magnitudes of blur.
2) A high magnitude of astigmatic blur resulted in higher error rates and error durations in the buzz-wire task, relative to a comparable magnitude of radially symmetric spherical blur. Low astigmatic blur with orthogonal axes in the two eyes produced higher error rates and error durations than comparable blur patterns with parallel axes in the two eyes. This effect is absent with high magnitudes of astigmatic blur.
3) Whereas similarly low levels of isometric and anisometric blur had similar effects on visuomotor performance, similarly high levels of isometric and anisometric blur did not. In particular, error durations were much greater with high levels of isometric blur than with anisometric blur.
4) The worsening of the error rate and error duration with optical blur was greater for binocular than monocular viewing conditions. This was reflected as an attenuation of the binocular advantage of task performance with low blur viewing and a complete loss of binocular advantage with high blur viewing, all relative to baseline no blur viewing.
5) The deterioration in buzz-wire task performance manifested differently across outcome variables in this study. The error duration (i.e. the percentage of total task time spent in error), was most sensitive to the presence of optical blur, whereas the speed was least sensitive.

Overall, these results support all but the third study hypothesis (see the heading Buzz-Wire Task Performance With Isometropia and Anisometropia for details). The results also agree with the previous literature that demonstrated losses in visuomotor task performance and prehensile movements with degraded binocularity arising from induced anisometropia,^13^^,^^20^ induced visibility loss through Bangerter foils,^22^ and in pathologies like keratoconus^4^ or amblyopia.^32^^,^^33^ The present study also extends these findings to other dimensions of blur (radial and interocular symmetry) that are hitherto absent in the literature to the best of the authors’ knowledge.

### Buzz-Wire Task Performance With Isometropia and Anisometropia

Interocular differences in blur magnitude (and/or axes) result in different retinal image qualities and/or aniseikonia, either of which may severely disrupt binocularity.^10^^,^^34^ This disruption of binocularity may have been responsible for our participants’ relatively poor performances in the buzz-wire task, when compared with what they were able to achieve with similiar blur magnitudes (and/or identical axes) in the two eyes. (see Figs. 1O, 1P to qualitatively experience this effect). Low magnitudes of spherical and astigmatic anisometropia also led to higher error duration relative to isometropia, even while this result did not reach statistical significance. Counterintuitively, isometropia led to greater task deterioration than anisometropia for high magnitudes of blur. This finding may be explained by the suppression of the blurred input in anisometropia, thus biasing the buzz-wire task toward the monocular performance of the eye with clear vision. This is suggested from the error duration with high anisometropia becoming similar to the baseline monocular viewing in Figure 5B of this study. This effect may also be observed qualitatively in Figure 1, wherein free-fusion of the simulated anisometropic image pair results in a clear cyclopean percept (see Figs. 1L, 1N), whereas free-fusion of simulated isometropic image pair results in a blurred cyclopean percept (see Figs. 1H, 1J). Indeed, this magnitude of anisometropia was found to induce suppression in the Piano and O'Connor study^13^ from which the blur values were chosen for the present study. Thus, even while the disparity signals may have become effectively useless, the monocular depth cues from the eye with clear vision could be reliably used to perform the buzz-wire task. In contrast, the visual system experiences a double whammy with high magnitude of isometropia – there is a loss of binocularity that negatively impacts stereopsis calculation and there is also a loss in spatial resolution that may preclude effective usage of the monocular depth cues. This monocular advantage may not be available in patients with anisometropia of different magnitudes of blur in the two eyes (e.g. high blur in one eye and low blur in the fellow eye). This condition was, however, not tested here given the already exhaustive list being investigated.

### Speed-Accuracy Trade-Off

Speed-accuracy trade-offs in motor tasks are usually assessed with a change in payoff matrix.^35^ If behavior changes with the payoff matrix, it may be due to a change in strategy, although one cannot rule out additional changes in perception. In the present study, changes in error rate (inverse of task accuracy) and speed with task difficulty may not necessarily reflect changes in response strategy, as the perception of the task itself changed with the different blurring lenses used in the study. In this context, a harder task can be expected to decrease response speed and/or increase the error rate. Only in 2 of our 15 participants, speed was positively correlated with error rate across the various blur conditions (*P* < 0.05). Consciously or unconsciously, these participants may have sacrificed accuracy to maintain speed across various blur conditions. Others did not show this correlation, indicating that sacrifices in speed or accuracy to optimize the complementary parameter is not a commonly observed phenomenon in the buzz-wire task.

### Clinical and Practical Implications of This Study

The present study was motivated by the previous observation of poorer buzz-wire task performance in individuals with keratoconus, relative to those with uncorrected myopia.^4^ The present results indicate that the combination of radial and interocular asymmetry of blur in keratoconus may have resulted in the greater loss of buzz-wire performance in this cohort, compared with their myopic counterparts. This observation, however, must be treated with caution, for the keratoconic cohort in the previous study were all corrected for their sphero-cylindrical refractive error. The retinal image quality of these participants may have thus be dominated by the radially asymmetric higher-order aberration terms (e.g. coma and trefoil^18^^,^^19^) and by any residual defocus and astigmatism that remained uncorrected. The present study did not induce blur from higher-order aberrations and thus its direct impact on the buzz-wire task performance remains unknown. Introduction of such patterns of blur before the eye is non-trivial, for it requires the use of advanced phase plates^36^^,^^37^ or adaptive optics devices,^38^ over a defined pupil size. Integration of such technology with visuomotor tasks is futuristic, at best.

The study has some practical implications for activities of daily living with blurred vision. Humans may perform visuomotor tasks with compromised vision that arises from their eye ailment (e.g. uncorrected refractive errors, cataract, and retinal pathology) or due to poor compliance in wearing their refractive correction. Whether or not visuomotor performance is impaired depends both on the task requirements and on the extent of vision loss. For instance, tasks that require only a gross judgment of depth may remain unimpaired in the presence of mild to moderate optical blur, whereas those that require finer depth judgments may be negatively impacted for comparable levels of blur. This is in line with the observations of Mann et al.^21^ wherein the degree of blur affected the interceptive tasks between a bat and a ball traveling at a certain speed in their study. Piano and O'Connor^13^ also observed that a water-pouring task requiring gross binocularity remained unimpaired with induced spherical anisometropic blur, whereas a bead-threading task (especially with smaller beads) requiring finer levels of binocularity was significantly impaired by comparable levels of blur. Clinicians are thus urged to consider the task requirements of their patients while planning the blur correction strategy (e.g. contact lens versus spectacle correction for certain sports activity) or counseling patients about their engagements in certain activities of daily living.^39^ As a corollary to this point, the study also recommends inclusion of a battery of functional vision tests that mimic routine activities of daily living with varying spatial and depth vision requirements. This may reduce the discordance often observed between the patient's clinical assessment that are largely based on measures of “sensory perception” (e.g. visual acuity, contrast sensitivity, and stereoacuity) and their ability to perform complex daily vision tasks. The latter tasks tend to challenge patients more than what may be expected from clinical vision testing.^40^^,^^41^

## Supplementary Material

Supplement 1
