# Supplementary material for: The Impact of Induced Optical Blur on Monocular and Binocular Depth-Related Visuomotor Task Performance
Source: Invest Ophthalmol Vis Sci. 2025 Dec 2;66(15):8. doi: 10.1167/iovs.66.15.8 (PMC12697702; doi:10.1167/iovs.66.15.8)
Supplement: Supplement 1 [file iovs-66-15-8_s001.pdf]

Supplementary information

Supplement I

Although high blur magnitudes ($6.25D\ x\ 45° \ and\ 8.75D\ x\ 45°$) produced performance deteriorations in the main experiment, low blur magnitudes ($2.25D\ x\ 45° \ and\ 3.25D\ x\ 45°$) did not show any evidence for being different. The first control experiment was conducted to put an upper bound on the magnitude of induced blur necessary for the buzz-wire task performance to significantly depart from the baseline no-blur viewing condition. Towards this end, 6 participants repeated the buzz-wire task (three of whom were already part of the main experiment) with the following magnitudes of isometropic spherical blur introduced before their eyes in randomized order: 1.25 D, 2.25 D, 3.25 D, 4.25 D, 5.25 D and 6.25 D. All other details were the same as the main experiment.

The averaged data of the 6 participants (Figure S1, top row) and the individual data of each participant (Figure 7, 2nd to 7th rows) shows a trend of increasing error rates (Figure S1, left column) and error duration (Figure S1, middle column) with increasing magnitudes of induced spherical blur in the binocular buzz-wire task. Speed did not appear to be altered with increase in the spherical blur (Figure S1, right column). One-factor RM-MANOVA showed a significant main effect of blur magnitude on the combined outcome variables ($p <0.001$). Univariate analyses confirmed that this effect was due to the worsening of the error rate ($p = 0.003$) and error duration ($p <0.001$). Post-hoc Bonferroni analyses revealed significant difference in performance between baseline viewing and with 6.25D of induced blur (error rate: $p = 0.04$; error duration: $p = 0.003$). No other pairwise comparisons reached statistical significance.

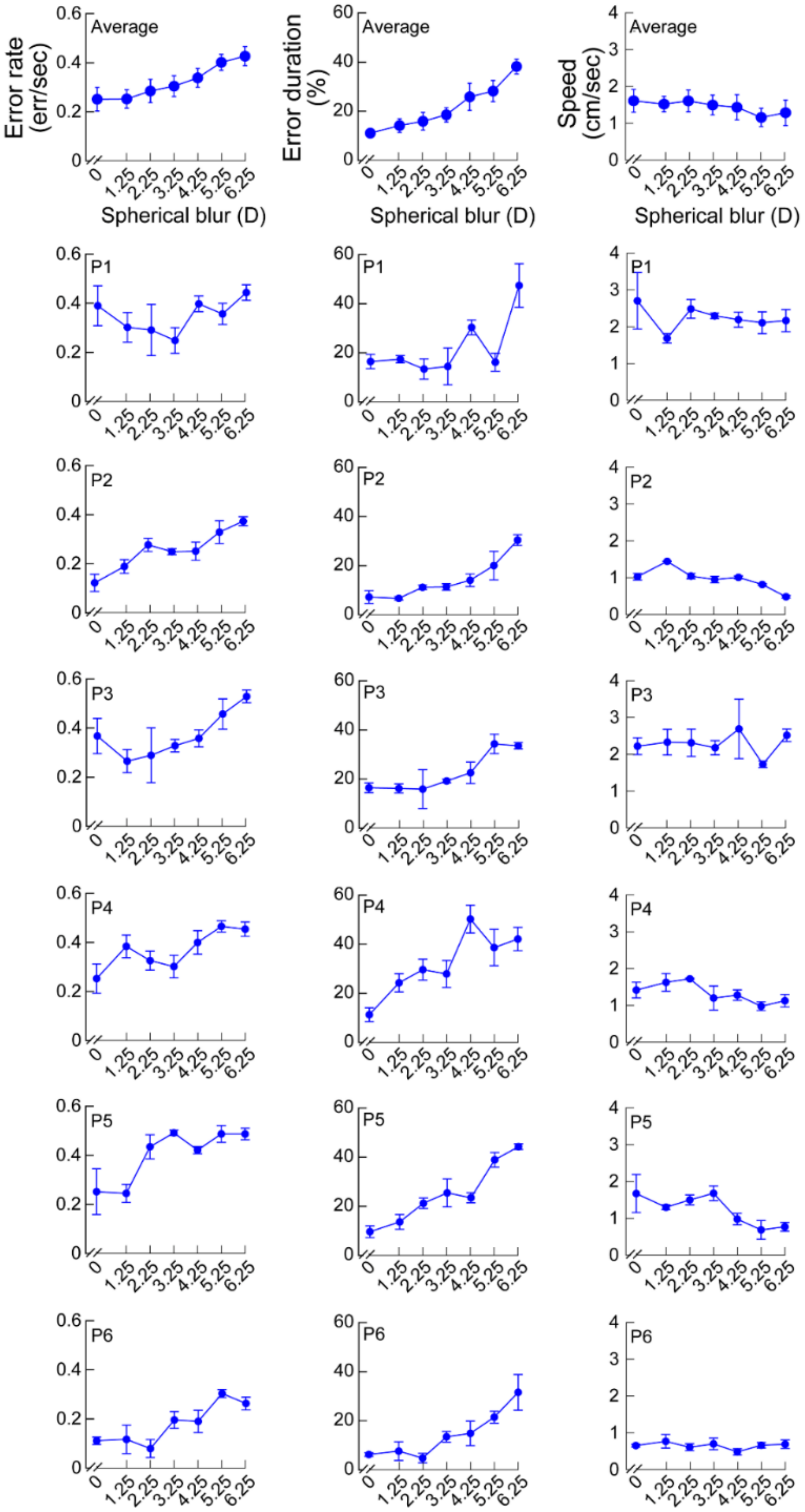

Figure S1: *Error rate (panel A), error duration (panel B) and speed (panel C) plotted as a function of the induced isometropic spherical blur in the first control experiment of this study. The top row shows average data across all participants, and the remaining rows show data from the individual participants. The lines connecting the dots are included only to help readers easily follow the data trends across different trials. The error bars in the top row represent the ±1 SEM across the mean data of all participants, while they represent the ±1 SEM across the three repeated trials in the individual participants.*

Supplement II

In the main experiment, each participant repeated the buzz-wire task 48 times, even while the order of the experimental conditions was randomized within and across participants. To address the potential impact of practice on the outcome measures, the second control experiment was performed wherein 6 new participants repeated the task 24 times each under binocular and monocular viewing conditions using the same buzz-wire patten. This task was performed with no additional blurring lenses. Participants P1, P2 and P3 performed the task binocularly first while participants P4, P5 and P6 performed the task monocularly first. The binocular and monocular versions of the task was performed on two separate days to avoid fatigue. All other details were the same as the main experiment.

Figure S2 plots the average (panel A) and individual (panels B – G) data of error rate (left column), error duration (middle column) and speed (right column) as a function of the trial number under binocular and monocular viewing conditions. The binocular data qualitatively showed a small improvement in error rate and error durations with increasing trials in the initial (Figure S2), but the 2-factor RM-MANOVA did not show any statistical significance in these trends. Inspection of the individual data reveals that this trend of improvement in task performance was present only in 3 subjects (P1, P2 and P3) and that too only under binocular viewing conditions of the initial trials. Binocular and monocular speed did not show any trend with increasing trial numbers (Figure S2). As expected, the univariate analysis showed statistically significant impact of viewing condition on all three outcome variables ($p \leq 0.007$, for all variables).

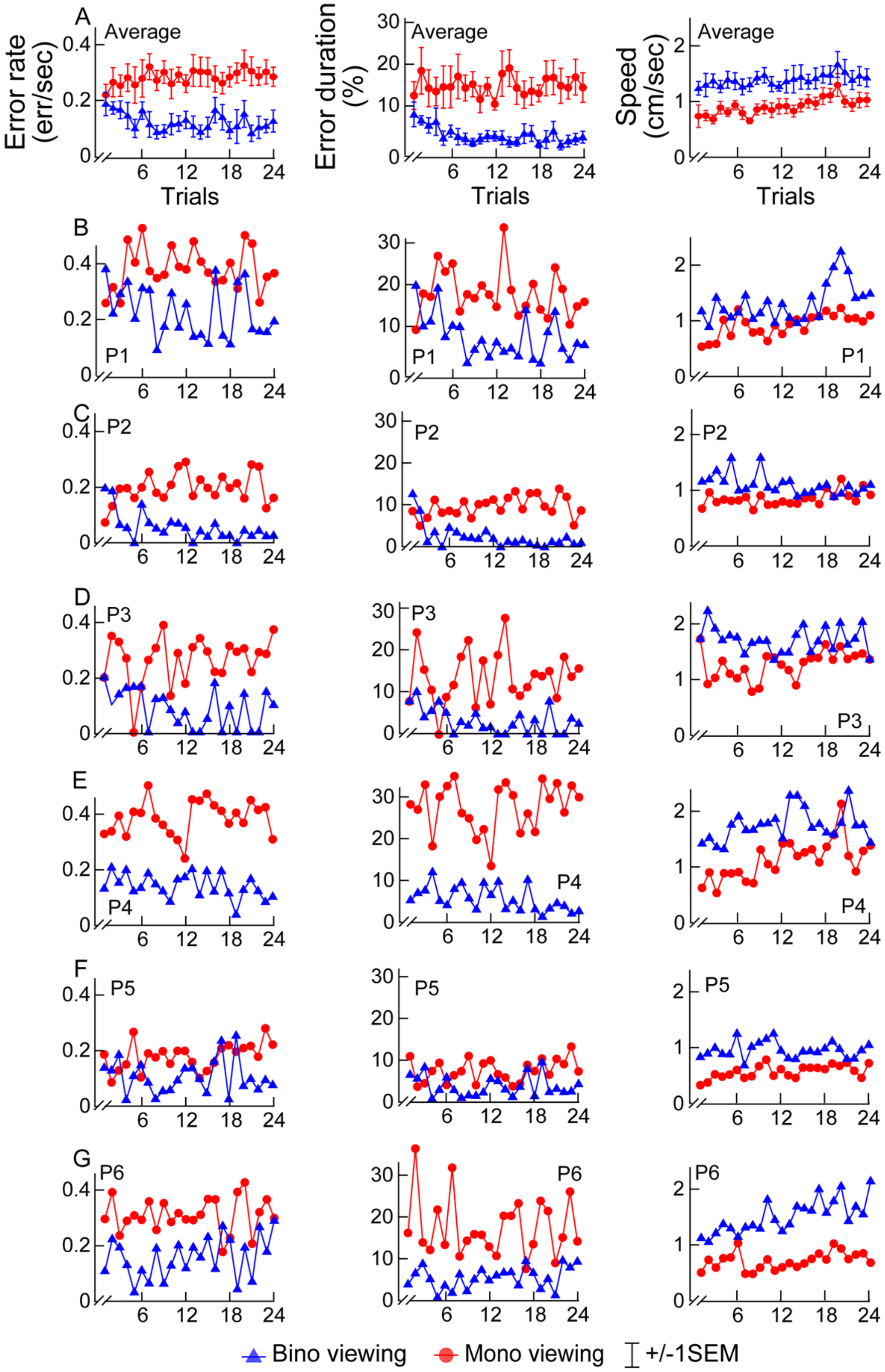

Figure S2: *Binocular and monocular error rate (left column), error duration (middle column) and speed (right column) plotted as a function of the repeated trials in the second control experiment of this study. The top row shows average data across all participants and the remaining rows show data from the individual participants. The error bars in the top row represents the ±1 SEM across the mean data of all participants across each trial.*
